# Hsa_circ_0000652 Aggravates Inflammation by Activation of Macrophages and Enhancement of OX40/OX40L Interaction in Ankylosing Spondylitis

**DOI:** 10.3389/fcell.2021.737599

**Published:** 2021-12-16

**Authors:** Minkai Song, Jiawen Gao, Tao Yan, Enguang Bi, Taixue An, Xiangyu Wang, Weizhou Jiang, Ting Wang, Zishuo Chen, Zhanjun Shi, Chao Zhang, Jun Xiao

**Affiliations:** ^1^ Division of Orthopaedic Surgery, Department of Orthopaedics, NanFang Hospital, Southern Medical University, Guangzhou, China; ^2^ Division of Spinal Surgery, Department of Orthopaedics, NanFang Hospital, Southern Medical University, Guangzhou, China; ^3^ Department of Biochemistry and Molecular Biology, School of Basic Medical Science, Guangdong Provincial Key Laboratory of Single Cell Technology and Application, Southern Medical University, Guangzhou, China; ^4^ Department of Laboratory Medicine, NanFang Hospital, Southern Medical University, Guangzhou, China; ^5^ Department of Endocrinology and Metabolism, NanFang Hospital, Southern Medical University, Guangzhou, China

**Keywords:** circular RNA, competing endogenous RNA, ankylosing spondylitis, macrophage activation, co-stimulatory molecules

## Abstract

Circular RNAs (circRNAs) have emerged as important roles in various inflammatory processes of rheumatic diseases. However, their expression profiles and influences in the pathogenesis of ankylosing spondylitis (AS) remain unclear. In this study, we revealed the differential expression profiles of circRNAs in peripheral blood mononuclear cells (PBMCs) in AS by circRNA sequencing. We screened the differentially expressed circRNAs in AS and verified that hsa_circ_0000652 was upregulated and had potential to be a biomarker of progression. Functionally, hsa_circ_0000652 promoted proliferation and cytokine production in macrophages and inhibited apoptosis. Through dual-luciferase assays and RNA pull-down assays, we demonstrated that hsa_circ_0000652 acted as a competing endogenous RNA (ceRNA) by binding with hsa-miR-1179 and regulated OX40L, which is characterized as a co-stimulatory molecule and found to be upregulated in AS patients. As a result, hsa_circ_0000652 aggravated the inflammation in the coculture system containing CD4^+^ T cells and macrophages via OX40/OX40L interaction. Our findings suggest that hsa_circ_0000652 was upregulated in AS patients and may serve as a pro-inflammatory factor in macrophages and a positive regulator of OX40/OX40L by sponging hsa-miR-1179.

## Introduction

Ankylosing spondylitis (AS) is one of the most common rheumatic diseases that cause general inflammation and irreversible arthritis involving the sacroiliac joints, hips, knees, and spinal joints (Taurog et al., 2016). Due to the poor understanding of pathogenesis in AS, there is still a lack of disease-specific molecular targets for diagnosis and treatment (Ward et al., 2019). Abnormal activation of immune cells is recognized as one of the leading causes in AS, but the underlying mechanisms and molecular regulators of AS disease have not been fully investigated (Menegatti et al., 2020). Consequently, there remains a need for detailed clarification of the molecular mechanism in inflammation progression of AS.

Extensive studies revealed that macrophages act as inflammatory mediators in many rheumatic diseases, including systemic lupus erythematosus (SLE) (Morel, 2017), rheumatoid arthritis (RA) (Tardito et al., 2019), and AS (Ranganathan et al., 2017a). Typically, macrophages are classified as pro-inflammatory macrophages (known as M1) and anti-inflammatory macrophages (known as M2). M1 macrophages, recognized as activated macrophages, which are common features in AS, are involved in bone formation (Horwood, 2016), cytokine production (Shapouri-Moghaddam et al., 2018), and T-cell activation (Guerriero, 2019). Therefore, it is necessary to further determine the mechanisms of macrophage activation in AS.

Circular RNA (circRNA) is well known because of its covalently looped structure by ligating the 3′ and 5′ terminals at the junction point (Pasman et al., 1996; Li X. et al., 2018). A massive number of studies showed that circRNAs are differentially expressed and functioned in various rheumatic and inflammatory diseases such as osteoarthritis (OA) (Shen et al., 2019), SLE (Li L.-J. et al., 2018), and RA (Yang et al., 2020). As a competing endogenous RNA (ceRNA), circRNA can act as a sponge for miRNA and regulate miRNA-related biological processes (Memczak et al., 2013). Unfortunately, the expression profiles and underlying ceRNA mechanisms of circRNAs in AS are rarely reported.

Accumulating data suggest that co-stimulatory molecules are critical in modulating the interactions of immune cells (Croft and Siegel, 2017; He et al., 2019). Among them, the OX40/OX40L axis is essential for CD4^+^ T-cell activation, and targeted therapy against OX40/OX40L is on a perspective way (Webb et al., 2016; Fu et al., 2020). Activated CD4^+^ T cells are the main effector cells in synovitis and enthesitis, which eventually result in inevitable damage of bones and joints in AS patients (Kidd et al., 1989; Watad et al., 2020). Basically, after the ligation of OX40/OX40L, both NF-kappa B and Akt/PI3K pathways are activated, leading to prolonged survival and augmented cytokine secretion (Song et al., 2004; Croft, 2010; Oh and Ghosh, 2013). Jacquemin et al. discovered that the OX40/OX40L axis downregulated Foxp3 expression in T cells and impaired the anti-inflammatory effect of regulatory T cells (Tregs) in SLE (Jacquemin et al., 2018). Despite the functions of the OX40/OX40L axis being clarified in many autoimmune diseases, the profiles of the OX40/OX40L axis in AS have not yet been reported.

In this study, we obtained a circRNA sequence to explore the differentially expressed circRNAs in peripheral blood mononuclear cells (PBMCs) from AS patients and identified a circRNA hsa_circ_0000652, which is upregulated in AS and correlated with disease activity. Subsequent assays revealed the role of hsa_circ_0000652 in macrophage activation and found that it could indirectly regulate OX40L expression by sponging hsa-miR-1179. We also discovered the OX40L^high^ cell population in AS patients. Coculture assay further revealed the enhanced OX40/OX40L interaction between CD4^+^ T cells and hsa_circ_0000652–expressing macrophages. Our findings suggested that hsa_circ_0000652 played a pro-inflammatory role in the inflammation of AS via macrophage activation and regulation of hsa-miR-1179/OX40L.

## Materials and Methods

### Human Samples and Ethical Approval

PBMCs used in this study were taken from 76 patients diagnosed with AS according to the modified New York 1984 criteria for AS and 40 healthy controls at NanFang Hospital of Southern Medical University, Guangzhou, China. Informed consents were obtained from patients before sample collection. The study was approved by the Medical Ethics Committee of NanFang Hospital of Southern Medical University. The collected PBMCs were isolated by using a lymphocyte separation medium (Solarbio, Beijing, China), following the manufacturer’s protocol for RNA extraction.

### CircRNA Sequencing

PBMCs from three recruited AS patients and three healthy donors were separated and used for high-throughput circRNA sequencing by Geneseed Biotech Co., Ltd. (Guangzhou, China). In brief, total RNA was extracted and purified using a Magen Hipure Total RNA Mini Kit (Magen, Guangzhou, China). After the construction of RNA sequencing libraries, a Qubit 3.0 fluorometer (Invitrogen, CA, United States) was used for quality control. Then the PE150 mode of HiSeq X10 (Illumina Inc., CA, United States) was used for sequencing. Differentially expressed circRNAs were screened, with a fold-change >1.5 and *p* < 0.05.

### Cell Culture, Differentiation, and Transfection

The human THP-1 and Jurkat T-cell lines were purchased from iCell Bioscience (Shanghai, China). THP-1 and Jurkat T cells were maintained in the RPMI-1640 medium (Gibco, MD, United States) with 15% fetal bovine serum (FBS, Gibco, MD, United States) and 1% penicillin–streptomycin solution (Gibco, MD, United States) in a humidified atmosphere with 5% CO_2_ at 37°C. The culture medium for THP-1 was supplemented with additional 50 μM of β-mercaptoethanol (Solarbio, Beijing, China). The THP1 cells were treated with 50 ng/ml phorbol 12-myristate 13-acetate (PMA, Solarbio, Beijing, China) for 24 h to induce THP1-derived M0 macrophages. For further differentiation of M1 macrophages, THP1-derived M0 macrophages were treated with 100 ng/ml lipopolysaccharides (LPS, Solarbio, Beijing, China) for 48 h.

The pLV-cir-cmv-mcs-EF1a-copGFP-puro plasmid used for lentivirus-mediated overexpression of hsa_circ_0000652 was obtained from Kidan Bioscience (Guangzhou, China). The pLKO.1-U6-EF1a-copGFP-T2A-puro plasmid cloned with short hairpin sequences–targeted hsa_circ_0000652 was purchased from IGE Biotechnology (Guangzhou, China) (Supplementary Table S2). The plasmid for overexpression or knockdown of hsa_circ_0000652 was co-transfected with pSPAX2 and pMD2.G into 293 T cells, respectively. The supernatant was collected and filtered after 48 h. THP-1 cells were cultured with the viral supernatant and supplemented with 5 μg/ml polybrene (Beyotime, Beijing, China). After 48 h, the cells were collected and re-seeded in a complete growth medium. To generate stably expressed cell lines, puromycin was then added to the culture medium for 7 days at the concentration of 1, 2, and 4 ug/ml in sequence. For miRNA transfection, mimics of hsa-miR-1179 and the control purchased from GeneCopoeia (Guangzhou, China) were transfected by Lipofectamine 3000 (Invitrogen, CA, United States).

For the coculture system, the stably expressed THP-1 cells were first seeded to 35-mm wells at the density of 2×10^5^ cells per well and differentiated to M1 macrophages as mentioned above. Jurkat T cells were then collected and added to each group of the macrophages at the density of 3.5×10^5^ cells per well for 24 h. To analyze the function of T cells, the suspension of the coculture system containing T cells was collected, washed with PBS, and added to new wells with a fresh complete growth medium. After a 24-h culture, the T cells were collected for RNA extraction and flow cytometry, while the supernatants were collected for cytokine measurement.

### RNA and Genomic DNA Extraction and Quantitative Real-Time PCR (qRT-PCR)

Total RNA from PBMCs and cultured cells was extracted using TRIzol reagent (Life Technologies, CA, United States) according to the manufacturer’s manuals. The RNA from the nucleus and cytoplasm was extracted using a PARIS kit (Life Technologies, CA, United States). The gDNA was extracted using a SteadyPure Universal Genomic DNA Extraction Kit (Accurate Biology, Hunan, China). RNA for mRNA and circRNA analysis was reverse-transcribed with Evo M-MLV RT Premix (Accurate Biology, Hunan, China) and used for qRT-PCR with Hieff qPCR SYBR Green Master Mix (Yeasen Biotechnology, Shanghai, China). RNA for miRNA analysis was reverse-transcribed and used for qRT-PCR using the an miDETECT A Track miRNA qRT-PCR Starter Kit (RiboBio, Guangzhou, China). The primers used for qRT-PCR are shown in Supplementary Table S1. Quantification of relative gene expression levels was calculated by using the 2^−ΔΔCT^ method with normalization using *U6* or *β-actin* as endogenous controls. The assay was performed in triplicate.

### RNase R Treatment

Total RNA extracted from THP-1–derived macrophages was incubated with or without RNase R (3 U/mg, Lucigen, WI, United States) for 30 min at 37°C. The treated RNA was then reverse-transcribed with Evo M-MLV RT Premix (Accurate Biology, Hunan, China). qRT-PCR of hsa_circ_0000652 and its linear host gene *IQGAP1* was conducted as described before.

### Fluorescence *In Situ* Hybridization (FISH)

The 6-FAM–labeled probe for hsa_circ_0000652 back-splice sequence and cy5-labeled probe for hsa-miR-1179 were synthesized by BersinBio (Guangzhou, China) (Supplementary Table S2). The circRNA FISH kit (BersinBio, Guangzhou, China) was then used for the next process. In brief, the probes were added to THP-1– derived macrophages permeabilized with 0.5% Triton X-100 and incubated in the hybridization buffer at 37°C overnight. After washing, the nuclei were stained with DAPI. The images captured by laser confocal microscopy were used to analyze the localization of hsa_circ_0000652 and hsa-miR-1179 in cells. Pearson’s correlation coefficient was used to quantify the co-localization.

### Cell Proliferation Assay

In the cell counting kit-8 (CCK-8, Beyotime, Beijing, China) assay, stably expressed THP-1 cells were seeded in 96-well plates with a density of 2×10^3^ per well and added with 10 μL of the CCK-8 reagent for 2 h at 0, 24, 48, 72, and 96 h. The optical density of CCK-8 at 450 nm was then measured using an Infinite 200 PRO microplate reader (TECAN, Switzerland). Results of each time point were analyzed with five independent replicates. In 5-ethynyl-2′-deoxyuridine (EdU) assay, stably expressed THP-1 cells were seeded in 12-well plates and derived to M0 macrophages as previously described. Then the cells were stained using the BeyoClick EdU-555 kit (Beyotime, Beijing, China) according to the manufacturer’s instructions. The nuclei of all the cells were stained with Hoechst 33,342. Images from three random scopes of each group captured by a fluorescence microscope were analyzed by ImageJ software (Bethesda, MD, United States).

### Cell Apoptosis Assay

The cell apoptosis assay for stably expressed THP-1 cells was conducted using the Annexin V-APC/PI Apoptosis Detection Kit (KeyGEN, Jiangsu, China) following the manufacturer’s instruction. In brief, the cells were collected and washed with 1× phosphate-buffered saline (PBS, Solarbio, Beijing, China), added with 3% FBS three times, and then incubated with Annexin V-APC/PI for 15 min. In the CytoFLEX flow cytometer (Beckman Coulter, CA, United States), the FL3 channel was used for detection of PI and FL4 channel was used to detect Annexin V-APC. The results were analyzed by CytExpert software (Beckman Coulter, CA, United States). The experiments were performed at least in triplicate.

### Flow Cytometry

To identify the expression patterns of OX40/OX40L on CD3^+^CD4^+^, CD3^+^CD4^−^, CD3^−^CD14^+^, CD3^−^CD19^+^, CD3^−^CD56^+^, and CD3^−^CD83^+^, PBMCs from recruited AS patients and healthy controls were collected for flow cytometry. The cells from one sample were separated to two tubes and washed with 1× phosphate buffer saline (PBS, Solarbio, Beijing, China) and added with 3% FBS three times. Then anti–CD3-PerCP 5.5, anti–CD4-PE Cy7, anti–CD14-FITC, anti–OX40-APC, and anti–OX40L-PE were added into the first tube of each sample. Anti–CD3-PerCP 5.5, anti–CD56-PE Cy7, anti–CD83-FITC, anti–CD19-APC Cy7, anti–OX40-APC, and anti–OX40L-PE were added into the second tube of each sample. Stably expressed THP-1 cells and Jurkat T cells from the coculture system were also stained with anti–OX40L-PE or anti–OX40-APC. All antibodies mentioned above were obtained from BioLegend (CA, United States). After incubation on ice for 30 min, the samples were washed and examined by using a CytoFLEX flow cytometer (Beckman Coulter, CA, United States). The data were analyzed using FlowJo software (Tree Star, OR, United States).

### Enzyme-Linked Immunosorbent Assay (ELISA)

The secretion levels of TNF-α, IL-6, and IL-23 in the supernatants of stably expressed THP-1–derived macrophages and the coculture system were measured by ELISA kits (MEIMIAN, Jiangsu, China) following the manufacturer’s instruction. The absorbance values at the wavelength of 450 nm were then measured using an Infinite 200 PRO microplate reader (TECAN, Switzerland). Data were expressed as mean ± standard deviation (SD) of three separate experiments.

### Dual-Luciferase Reporter Assay

The wild-type (WT) and mutant (Mut) sequences of the predicted binding sites of hsa-miR-1179 in hsa_circ_0000652 were first cloned to the pEZX-MT06 luciferase vector (GeneCopoeia, Guangzhou, China) (Supplementary Table S2). The empty vector was used as a negative control (NC). Then the vectors were co-transfected with the hsa-miR-1179 mimic or its negative control into 293 T cells using Lipofectamine 3000 (Invitrogen, CA, United States), respectively. Activity of Renilla luciferase and firefly luciferase was determined at 48 h after transfection using the Luc-pair Duo-Luciferase HS assay kit (GeneCopoeia, Guangzhou, China).

### RNA Pull-Down Assay

Biotin-labeled probes for hsa_circ_0000652, hsa-miR-1179, and corresponding negative control were designed and synthesized by RiboBio (Guangzhou, China) (Supplementary Table S2). The RNA pull-down kit (BersinBio, Guangzhou, China) was used for the assay according to the manufacturer’s protocol. In brief, the biotin-labeled probes were first conjugated with magnetic beads for 30 min. Then the cells were cross-linked with formaldehyde and lysed and incubated with magnetic beads at 4°C overnight. After treatment with proteinase K, the samples were added with TRIzol reagent and used for RNA extraction as previously described. QRT-PCR was used to analyze the expression of hsa-miR-1179, hsa_circ_0000652, and OX40L mRNA, respectively.

### Statistical Analysis

Each experiment was conducted with at least three independent replicates. The quantitative data with Gaussian distribution were presented as mean ± SD, and the data with non-Gaussian distribution were presented as median with quartile. Student’s *t-*test, Welch’s *t*-test, or the Mann–Whitney *U* test was used for comparison of the two groups depending on distribution and homoscedasticity of data. Two-way ANOVA was used for multi-factorial comparisons. Pearson’s correlation was used to identify the correlation. All tests were performed and calculated using GraphPad Prism 8 (GraphPad Software, CA, United States). A *p* value <0.05 was considered statistically significant.

## Results

### Characteristics of Differentially Expressed circRNAs in AS

**FIGURE 1:**
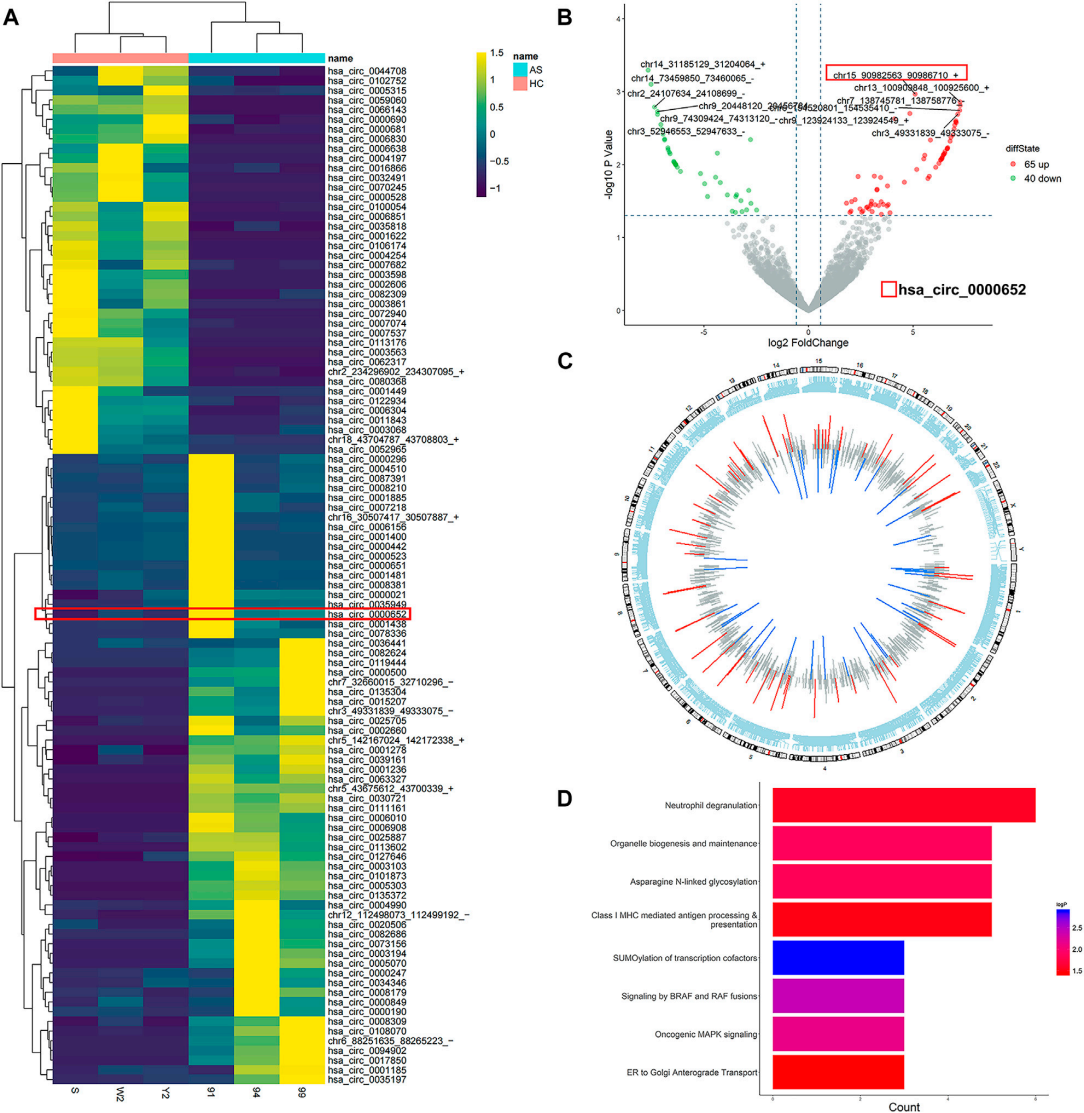
Characteristics of differentially expressed circRNAs in PBMCs from AS patients. **(A)** Heatmap of all differentially expressed circRNAs in PBMCs from AS patients; **(B)** volcano scatter plot of differentially expressed circRNAs in PBMCs from AS patients; **(C)** chromosome distribution of differentially expressed circRNAs in PBMCs from AS patients; **(D)** reactome enrichment dot plot of the host genes of differentially expressed circRNAs in PBMCs from AS patients.

To explore the differentially expressed circRNAs in AS, three samples of PBMCs from AS patients with high disease activity (ASDAS_CRP_ score >2.1) and three samples from healthy controls were analyzed by high-throughput circRNA sequencing. With the threshold of fold-change > 1.5 and *p* value <0.05, we identified 65 upregulated and 40 downregulated circRNAs in AS patients compared to healthy controls (Figures 1A,B). Then we further analyzed chromosome distribution of differentially expressed circRNAs and have presented them in Figure 1C. Reactome enrichment was used to identify the biological pathways, indicating that multiple inflammatory pathways were involved in AS (Figure 1D). Accordingly, these results showed that the expression profiles of multiple circRNAs varied in AS patients and healthy controls and may be correlated with inflammatory pathways.

### Hsa_circ_0000652 Is Upregulated in PBMCs From AS Patients and Associated With Disease Activity

**FIGURE 2:**
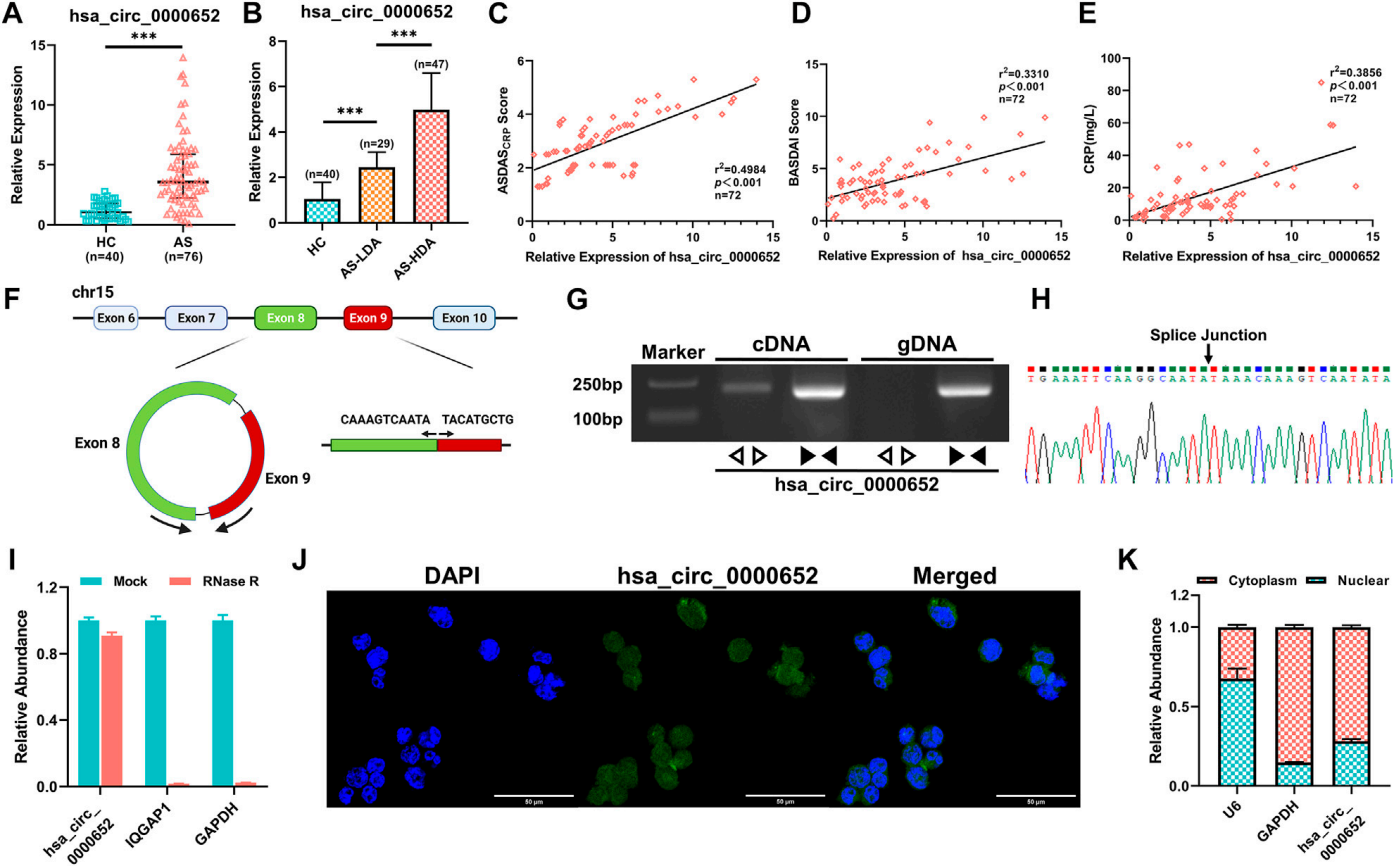
Hsa_circ_0000652 is upregulated in PBMCs from AS patients and correlated with disease activity. **(A–B)** Expression of hsa_circ_0000652 in PBMCs from 76 AS patients with different disease activities and 40 healthy controls was analyzed by quantitative real-time PCR (qRT-PCR). **(C–E)** Correlation analysis for the expression of hsa_circ_0000652 with the Ankylosing Spondylitis Disease Activity Score (ASDAS_CRP_), Bath Ankylosing Spondylitis Disease Activity Index (BASDAI), and C-reactive protein (CRP) level (mg/L) of AS patients (n = 72). **(F)** Schematic illustration showing the formation of hsa_circ_0000652 from its host gene *IQGAP1* by back-splicing. **(G)** Agarose gel electrophoresis of PCR amplification by divergent and convergent primers of hsa_circ_0000652 in cDNA and gDNA. **(H)** Back-splice junction point of hsa_circ_0000652 was identified by Sanger sequencing. **(I)** qRT-PCR analysis of hsa_circ_0000652 and *IQGAP1* and *GAPDH* with or without RNase R treatment (n = 3). **(J–K)** FISH assay and nuclear-cytoplasmic separation assay showed that hsa_circ_0000652 was mainly localized in the cytoplasm of the THP-1 cell line. Data in A and B are presented as median ± quartile and data in I and H are presented as mean ± SD. ***p < 0.001.

Among the differentially expressed circRNAs, hsa_circ_0000652 was significantly upregulated in 76 AS patients compared to 40 healthy controls by qRT-PCR analysis (Figure 2A, *p* < 0.001). Interestingly, the expression levels of hsa_circ_0000652 in AS patients with low disease activity (AS-LDA, 1.3 < ASDAS_CRP_ score ≤2.1) were still significantly higher than those in healthy controls and lower than those in patients with severe disease activity (AS-HDA, ASDAS_CRP_ score >2.1) (Figure 2B, n of HC = 40, n of AS-LDA = 29, and n of AS-HDA = 47, *p* < 0.001 for AS-LDA vs. HC and AS-HDA vs AS-LDA). Correlation analysis suggested that the expression levels of hsa_circ_0000652 were statistically correlated with the Ankylosing Spondylitis Disease Activity Score (ASDAS_CRP_), Bath Ankylosing Spondylitis Disease Activity Index (BASDAI), and level of C-reactive protein (Figures 2C–E, *p* < 0.001).

Bioinformatics analysis implicated that hsa_circ_0000652 was formed by exons 8 and 9 of its host gene *IQGAP1* by back-splicing (Figure 2F). To verify the formation of hsa_circ_0000652, we performed PCR amplification and agarose gel electrophoresis for cDNA and gDNA using divergent and convergent primers. The results showed that convergent primers were amplified only in cDNA while divergent primers could be amplified in both kinds of the DNA template (Figure 2G). Sanger sequencing of the PCR product amplified by divergent primers identified the back-splice junction point of hsa_circ_0000652 (Figure 2H). After RNase R digestion, linear RNAs such as *GAPDH* and *IQGAP1* were degraded, but hsa_circ_0000652 was still intact due to its circular structure (Figure 2I). Notably, FISH assay and nuclear-cytoplasmic separation, followed by qRT-PCR analysis suggested that hsa_circ_0000652 was mainly localized in the cytoplasm (Figures 2J,K, Pearson’s R value = 0.59 for co-localization in FISH assay of hsa_circ_0000652 and DAPI).

### Hsa_circ_0000652 Modulates Activation of Macrophages

**FIGURE 3:**
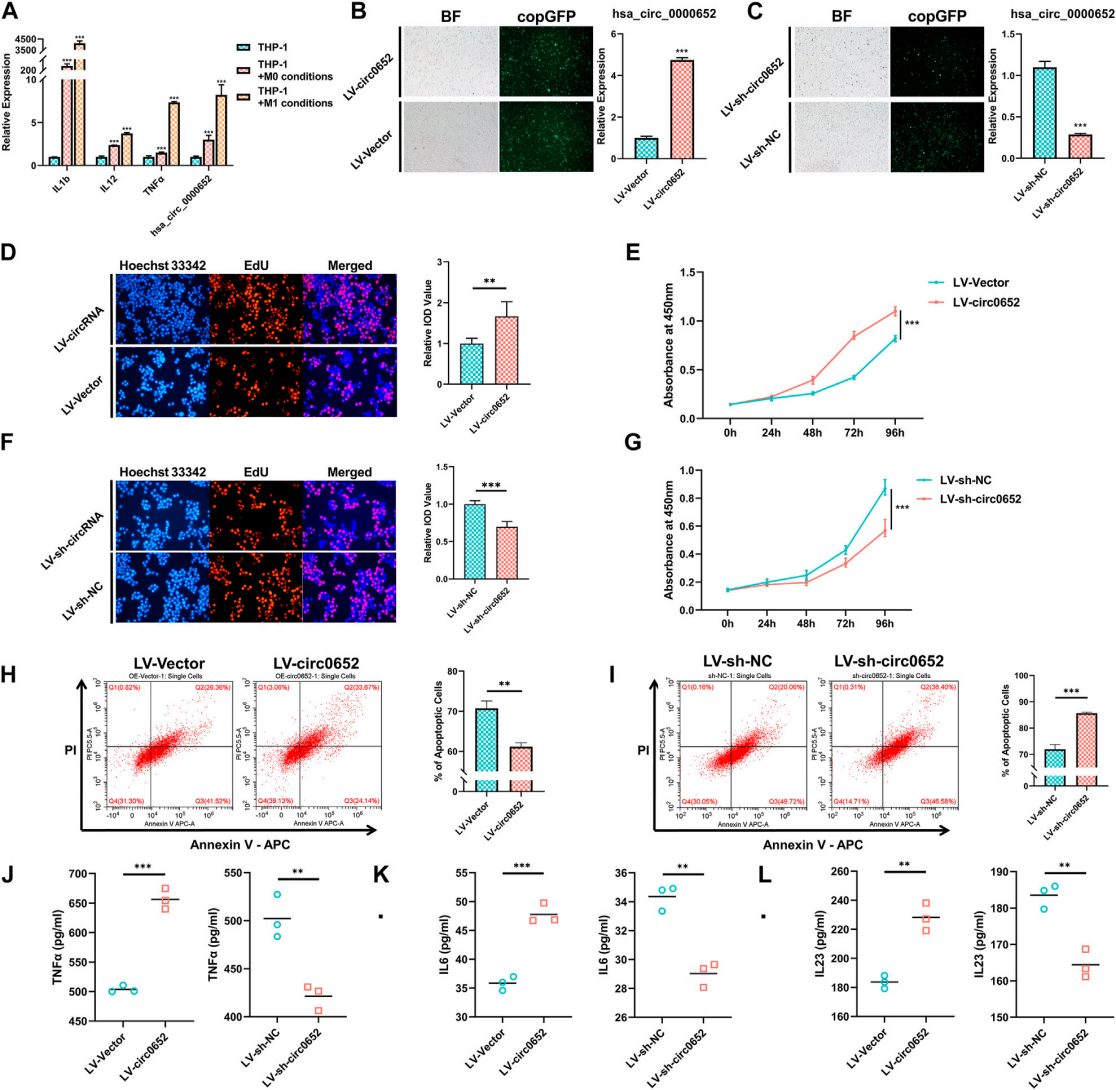
Hsa_circ_0000652 promotes the activation of macrophages. **(A)** Quantitative real-time PCR (qRT-PCR) analysis of IL-1b, IL-12, TNF-α, and hsa_circ_0000652 during the activation of THP-1–derived macrophages (n = 3); **(B–C)** observation of copGFP and qRT-PCR analysis in hsa_circ_0000652 stably expressed THP-1 cell lines; **(D–G)** 5-ethynyl-2′-deoxyuridine (EdU) assay and cell counting kit-8 (CCK-8) assay after overexpression and knockdown of hsa_circ_0000652; **(H–I)** Annexin V–PI assay determined the apoptotic cell ratio of THP-1–derived macrophages with overexpression and knockdown of hsa_circ_0000652 (n = 3); **(J–L)** ELISA of cytokine levels in the culture medium of THP-1–derived macrophages with overexpression and knockdown of hsa_circ_0000652 (n = 3). Data in bar plots and line plots are presented as mean ± SD, and data in scatter plots are supplemented with line of mean. **p < 0.01; ***P < 0.001. Each experiment was performed at least in triplicate.

As antigen-processing cells have important roles in AS, we hypothesized that hsa_circ_0000652 participated in mononuclear macrophage–related inflammation. qRT-PCR showed that the expression levels of hsa_circ_0000652 and marker genes related to macrophage activation significantly increased in THP-1–derived M0 and M1 macrophages compared to those in wild-type THP-1 (Figure 3A, TNF-α: *p* = 0.0053 for M0 vs. wild-type; hsa_circ_0000652: *p* = 0.0024 for M0 vs. wild-type, *p* = 0.0022 for M0 vs. M1; others: *p* < 0.001 for M0 vs. wild-type and M1 vs. M0). To further determine the functions of hsa_circ_0000652, we constructed stably expressed THP-1 cell lines with overexpression or knockdown of hsa_circ_0000652 (Figures 3B,C, *p* < 0.001 for LV-Vector vs. LV-circ0652; *p* = 0.0019 for LV-sh-NC vs. LV-sh-circ0652).

Proliferation and apoptosis are closely related with the activation of macrophages. The results of EdU and CCK-8 assays illustrated that THP-1–derived macrophages with overexpression of hsa_circ_0000652 (LV-circ0652) had a significantly higher ratio of proliferating cells than its negative controls (LV-Vector) (Figures 3D,E, *p* < 0.001). But, the knockdown of hsa_circ_0000652 (LV-sh-circ0652) led to a restraint of proliferation compared to controls (LV-sh-NC) (Figures 3F,G, *p* < 0.001). Flow cytometry assay demonstrated that THP-1 cells derived from macrophages with overexpression of hsa_circ_0000652 exhibited a lower ratio of apoptoptic cells than that of control cells, while knockdown of hsa_circ_0000652 significantly increased the amount of apoptoptic cells (Figures 3H,I, *p* = 0.0013 for LV-Vector vs. LV-circ0652; *p* < 0.001 for LV-sh-NC vs. LV-sh-circ0652).

ELISA analysis for pro-inflammatory cytokines demonstrated that TNF-α, IL-6, and IL-23 in the culture medium of THP-1–derived macrophages with overexpression of hsa_circ_0000652 were elevated compared to those of the control (Figure 3J-L, TNF-α and IL-6: *p* < 0.001, IL-23: *p* = 0.0018 for LV-Vector vs. LV-circ0652). Meanwhile, knockdown of hsa_circ_0000652 ameliorated the secretion of TNF-α, IL-6, and IL-23 compared to that of control cells (Figure 3J-L, TNF-α: *p* = 0.0058, IL-6: *p* = 0.0016, IL-23: *p* = 0.0030 for LV-sh-NC vs. LV-sh-circ0652). In summary, hsa_circ_0000652 modulated macrophage activation by regulating proliferation, apoptosis, and secretion of pro-inflammatory cytokines.

### Hsa_circ_0000652 Serves as a Sponge of Hsa-miR-1179 to Upregulate OX40L Expression

**FIGURE 4:**
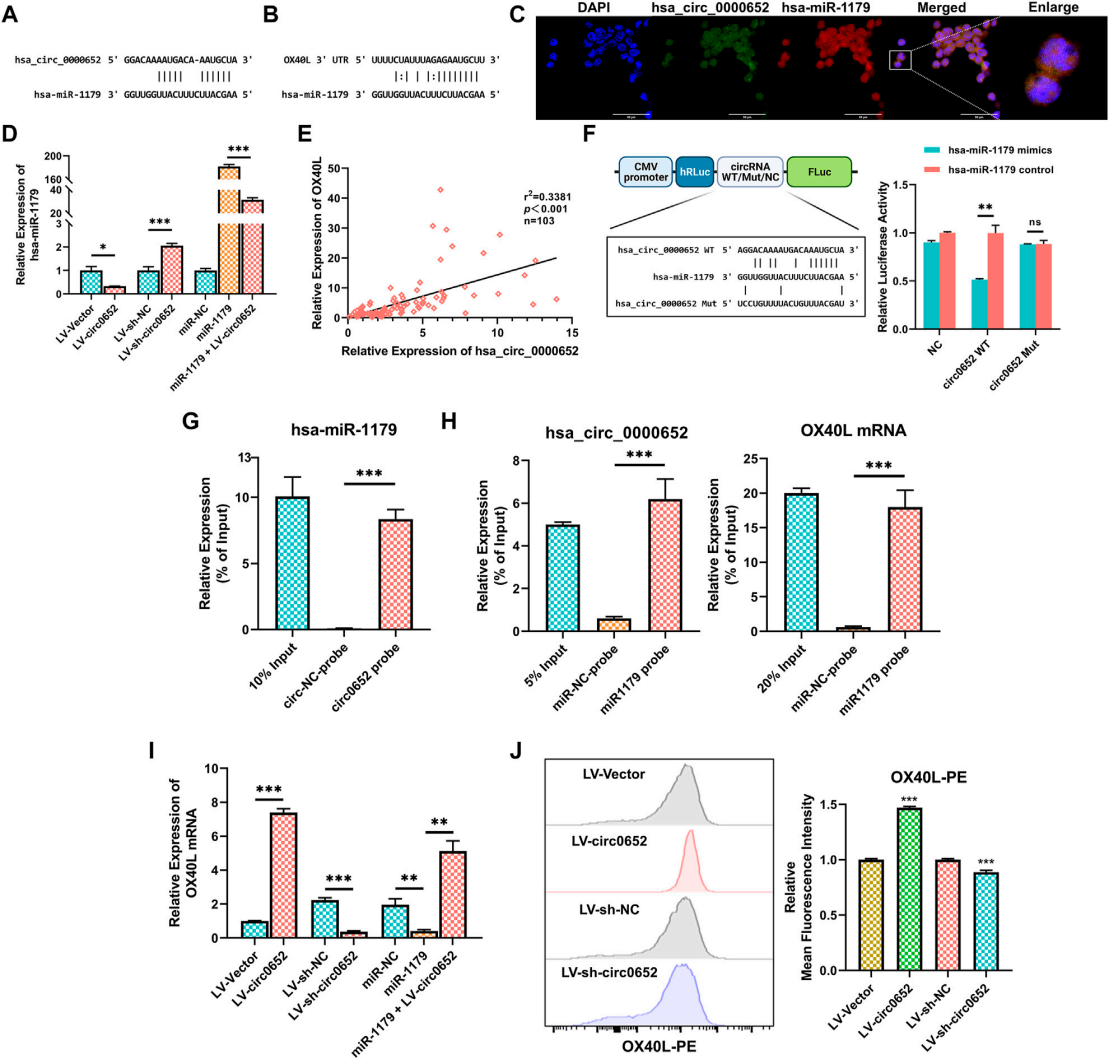
Hsa_circ_0000652 upregulates OX40L expression by sponging hsa-miR-1179. **(A–B)** Binding sites of hsa_circ_0000652 and hsa-miR-1179 and 3′ UTR of OX40L mRNA and hsa-miR-1179 predicted by circInteractome, TargetScan, and MiRanda platforms. **(C)** Co-localization analysis of hsa_circ_0000652 and hsa-miR-1179 in THP-1–derived macrophages by fluorescence *in situ* hybridization (FISH) assay. **(D)** Quantitative real-time PCR (qRT-PCR) analysis of hsa-miR-1179 in hsa_circ_0000652 stably expressed THP-1 cells and THP-1 transfected with the hsa-miR-1179 mimic or negative control (n = 3). **(E)** Correlation analysis for hsa_circ_0000652 and OX40L mRNA in PBMCs from AS patients and healthy controls (n = 103). **(F)** Dual-luciferase assay detected the binding between hsa_circ_0000652 and hsa-miR-1179. **(G)** qRT-PCR analysis of hsa-miR-1179 in the immunoprecipitates of RNA immunoprecipitation (RIP) assay using probes for hsa_circ_0000652 or the negative control (n = 3). **(H)** qRT-PCR analysis of hsa_circ_0000652 and OX40L mRNA in the immunoprecipitates of RIP assay using probes for hsa-miR-1179 or the negative control (n = 3). **(I)** QRT-PCR analysis of OX40L in hsa_circ_0000652 stably expressed THP-1 cells and THP-1 transfected with hsa-miR-1179 mimic or the negative control (n = 3). **(J)** Flow cytometry analysis of OX40L on hsa_circ_0000652 stably expressed THP-1 cells (n = 3). Data in bar plots are presented as mean ± SD. *p < 0.05; **p < 0.01; ***p < 0.001.

To further investigate the underlying functions of hsa_circ_0000652 in macrophages, bioinformatics analysis was conducted. The results from circInteractome, TargetScan, and MiRanda platform predicted that hsa_circ_0000652 potentially served as a sponge for hsa-miR-1179, while hsa-miR-1179 could bind to 3′-UTR of OX40L mRNA (Figures 4A,B). Moreover, FISH assay demonstrated that hsa_circ_0000652 and hsa-miR-1179 co-localized in THP-1–derived macrophages (Figure 4C, Pearson’s R value = 0.81 for co-localization of hsa_circ_0000652 and hsa-miR-1179). Interestingly, the expression of hsa-miR-1179 was downregulated in hsa_circ_0000652 overexpressed cells but upregulated in hsa_circ_0000652 knockdown cells (Figure 4D, *p* = 0.0178 for LV-Vector vs. LV-circ0652; *p* < 0.001 for LV-sh-NC vs. LV-sh-circ0652). Besides, hsa-miR-1179 was decreased in cells co-infected with hsa_circ_0000652 and hsa-miR-1179 compared to cells transfected with hsa-miR-1179 only (Figure 4D, *p* < 0.001). Furthermore, the expression of hsa_circ_0000652 was found to be significantly correlated with OX40L expression in PBMCs (Figure 4E, *p* < 0.001). Based on these results, we hypothesized that hsa_circ_0000652 could indirectly upregulate OX40L expression by blocking hsa-miR-1179.

In the dual luciferase reporter assay, the hsa-miR-1179 mimic remarkably attenuated the luciferase activity of cells co-transfected with wild-type hsa_circ_0000652 but did not change in the mutant group (Figure 4F, *p* = 0.0081 for circ0652 WT + hsa-miR-1179 mimic vs. circ0652 WT + NC mimic, *p* = 0.8290 for circ0652 Mut + hsa-miR-1179 mimic vs. circ0652 Mut + NC mimic). In the RNA pull-down assay, qRT-PCR analysis showed that more hsa-miR-1179 were enriched by hsa_circ_0000652 probes than by control probes (Figure 4G, *p* < 0.001 for the circ0652 probe vs. the circ-NC probe). Moreover, biotin-labeled hsa-miR-1179 probes enriched more hsa_circ_0000652 and OX40L mRNA than control probes (Figure 4H, *p* < 0.001 for miR1179 probe vs. miR-NC probe).

To clarify the indirect regulation between hsa_circ_0000652 and OX40L, we performed qRT-PCR and flow cytometry of OX40L in hsa_circ_0000652 stably expressed THP-1 cells. Notably, the expression levels of OX40L mRNA were higher in cells with hsa_circ_0000652 overexpression than the control but lower in cells with hsa_circ_0000652 knockdown (Figure 4I, *p* < 0.001 for LV-Vector vs. LV-circ0652 and LV-sh-NC vs. LV-sh-circ0652). THP-1 cells transfected with hsa-miR-1179 mimics also showed reduced OX40L mRNA expression than the control (Figure 4I, *p* = 0.0016 for miR-1179 vs. miR-NC). Remarkably, the reduction was partially rescued in cells co-infected with hsa-miR-1179 and hsa_circ_0000652 (Figure 4I, *p* = 0.0048 for miR-1179 vs. miR-NC). In flow cytometry analysis, the expression of OX40L was significantly greater in cells with overexpression of hsa_circ_0000652 than in controls (Figure 4J, *p* < 0.001 for LV-Vector vs. LV-circ0652). Meanwhile, knockdown of hsa_circ_0000652 resulted in attenuated expression of OX40L than controls (Figure 4J, *p* < 0.001 for LV-sh-NC vs. LV-sh-circ0652). Collectively, the results described above revealed that hsa_circ_0000652 upregulated the expression of OX40L by blocking hsa-miR-1179.

### Expression Profiles of OX40L in AS

**FIGURE 5:**
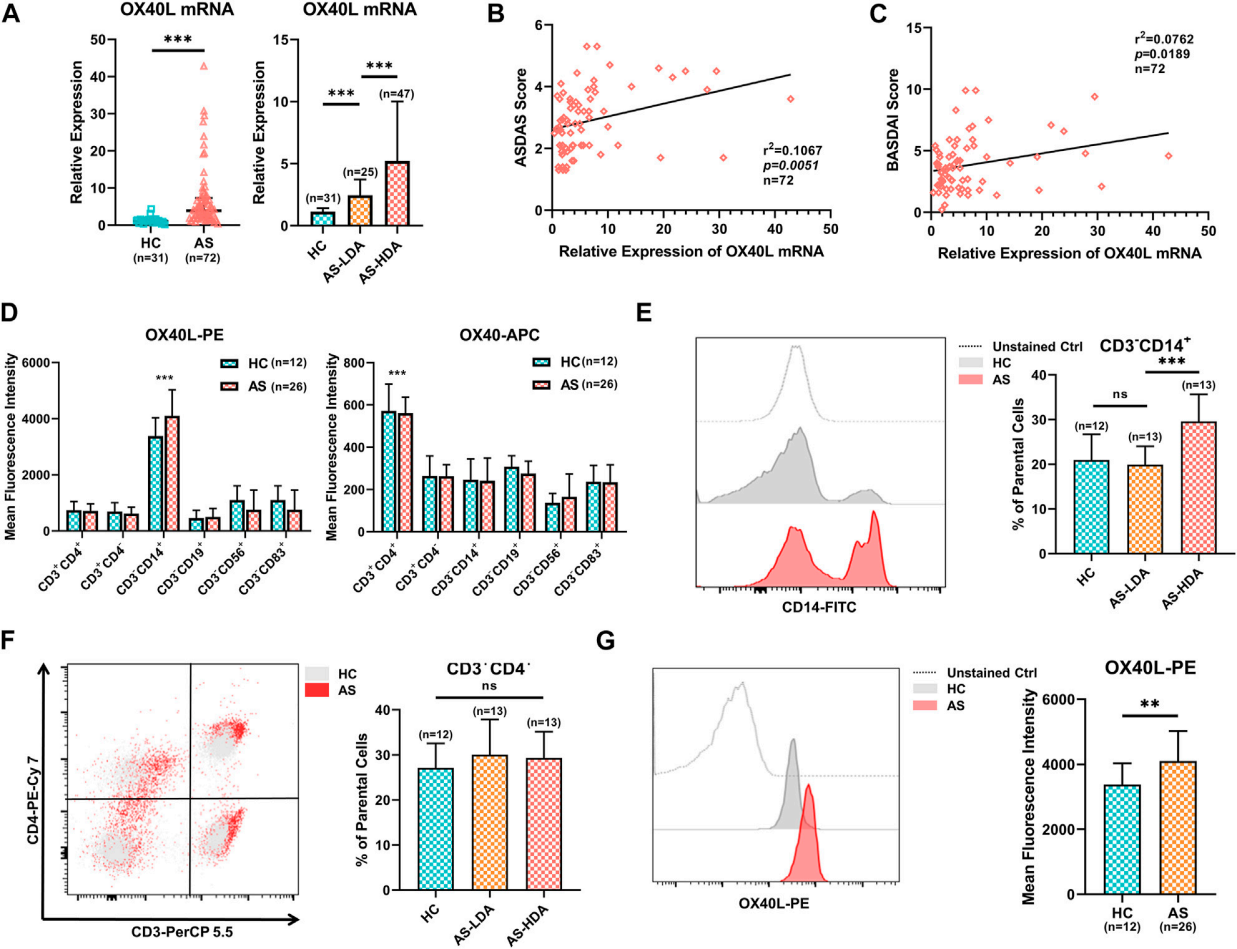
Expression profiles of OX40L in PBMCs of AS patients. **(A)** Quantitative real-time PCR (qRT-PCR) analysis of OX40L mRNA in PBMCs of 72 AS patients with different disease activities and 31 healthy controls. **(B,C)** Correlation analysis for OX40L in PBMCs with the ASDAS and BASDAI score of AS patients (n = 72). (D). OX40L and its receptor, OX40, were tested in PBMCs of AS patients (n = 26) and healthy controls (n = 12) by flow cytometry. Mean fluorescence intensity (MFI) was used to analyze differentially expressed OX40/OX40L on cells marked by CD3^+^CD4^+^, CD3^+^CD4^−^, CD3^−^CD14^+^, CD3^−^CD19^+^, CD3^−^CD56^+^, and CD3^−^CD83^+^. **(E,F)** Cell ratios of CD3^−^CD14^+^ cells and CD3^+^CD4^+^ T cells in PBMCs of AS patients (n = 26) and healthy controls (n = 12) were detected by flow cytometry; **(G)** represented illustration and MFI of OX40L on the CD3^−^CD14^+^ cell group of PBMCs from AS patients and healthy controls followed by flow cytometry. Data in A are presented as median ± quartile, and data in other chart plots are presented as mean ± SD. ns: *p* ≥ 0.05; **p < 0.01; ***p < 0.001.

As the expression of OX40L was not yet reported in AS, we performed several assays on PBMCs obtained from AS patients and healthy controls. First, qRT-PCR analysis demonstrated that increased OX40L expression was seen in the AS group compared with that of the healthy controls (left of Figure 5A, *p* < 0.001). Moreover, the expression of OX40L in AS patients with low disease activity was significantly higher than that of the healthy controls and lower than that in patients with high disease activity (right of Figure 5A, *p* < 0.001 for AS-HDA vs. AS-LDA and AS-LDA vs. HC). Furthermore, the ASDAS score and BASDAI score of involved AS patients were found to be correlated with the expression level of OX40L (Figures 5B,C, *p* < 0.001).

OX40L is widely expressed on multiple antigen-processing cells with various functions. To reveal the high OX40L-expressing cells, we analyzed the expression of OX40L in different cell populations of PBMCs from AS patients and healthy controls by flow cytometry and found that OX40L was mainly expressed by CD3^−^CD14^+^ cells, while its receptor OX40 was mainly located on CD3^+^CD4^+^ T cells (Figure 5D, *p* < 0.001 for OX40L on CD3^−^CD14^+^ cells and OX40 on CD3^+^CD4^+^ T cells compared to other cell groups). Further analysis showed that the percentage of CD3^−^CD14^+^ cells was remarkably higher in AS patients with high disease activity than patients with low disease activity and healthy controls (Figure 5E, *p* < 0.001 for AS-HDA vs. AS-LDA, *p* = 0.6211 for AS = LDA vs. HC). Nevertheless, cell ratios of CD3^+^CD4^+^ T cells did not differ among groups of AS patients with high or low disease activity and healthy controls (Figure 5F, *p* = 0.7931 for AS-HDA vs. AS-LDA, *p* = 0.2911 for AS = LDA vs. HC). Additionally, OX40L expression on CD3^−^CD14^+^ cells was increased in AS patients, rather than healthy controls (Figure 5G, *p* = 0.0067). Taken together, OX40L was upregulated and correlated with disease activity in AS patients and is mainly expressed on CD3^−^CD14^+^ cells.

### Hsa_circ_0000652 Enhances the Interaction Between Macrophages and CD4^+^ T Cells via OX40/OX40L

**FIGURE 6:**
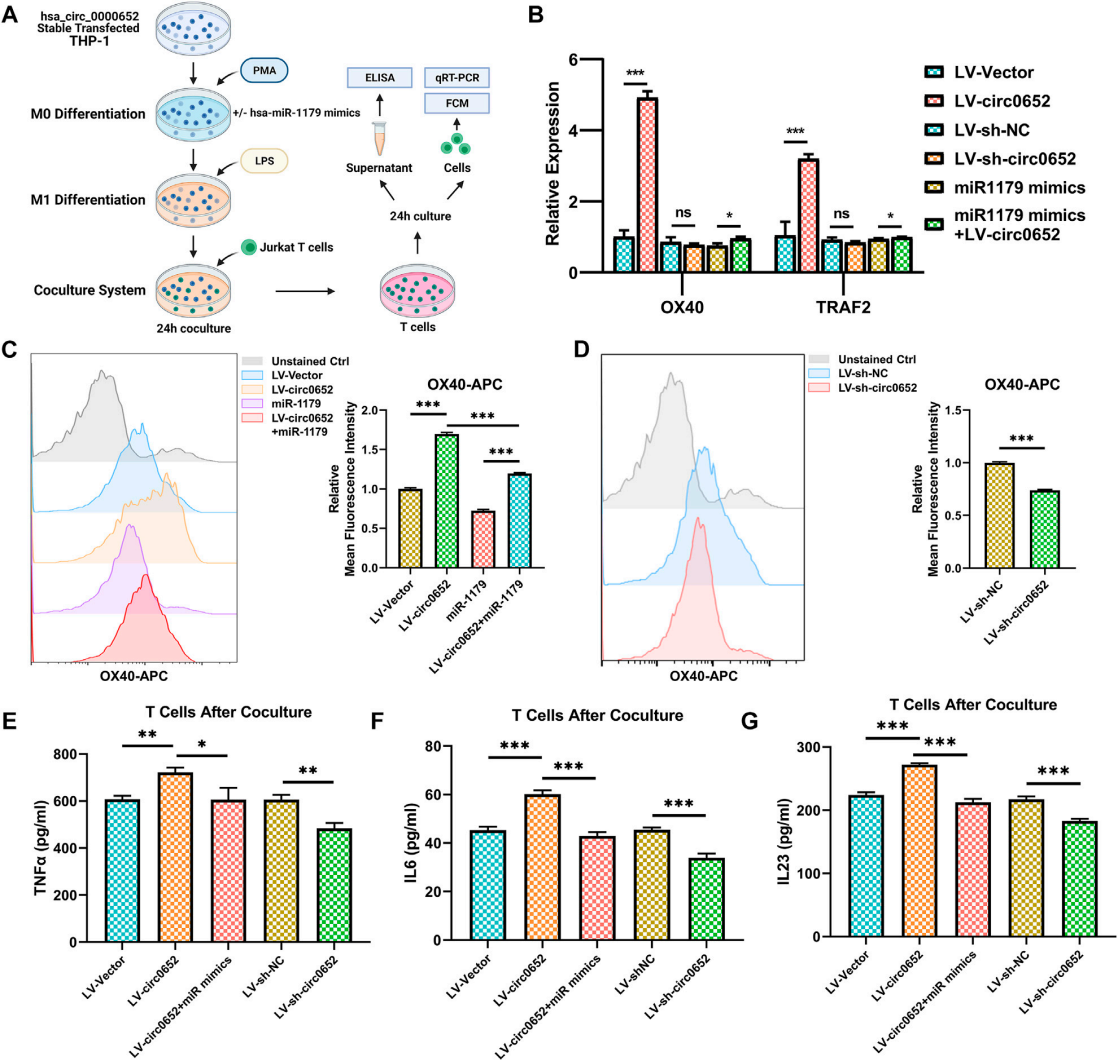
Hsa_circ_0000652 regulates inflammation caused by OX40/OX40L interaction between macrophages and CD4^+^ T cells. **(A)** Schematic illustration of macrophages and CD4^+^ T-cell coculture system. **(B)** QRT-PCR analysis of OX40 and OX40L in CD4^+^ T cells after being cocultured with hsa_circ_0000652 stably expressed THP-1 cell lines with or without hsa-miR-1179 mimics (n = 3). **(C,D)** Flow cytometry of OX40 in CD4^+^ T cells after coculturing with hsa_circ_0000652 stably expressed THP-1 cell lines with or without hsa-miR-1179 mimics (n = 3). **(E–G)** ELISA analysis of cytokine levels in the supernatant of CD4^+^ T cells after coculturing with hsa_circ_0000652 stably expressed THP-1–derived macrophages with or without transfection of hsa-miR-1179 (n = 3). Data are presented as mean ± SD. ns: *p* ≥ 0.05; *p < 0.05; **p < 0.01; ***p < 0.001. Each experiment was performed at least in triplicate.

To verify the effect of OX40/OX40L interaction regulated by hsa_circ_0000652, we designed a coculture system of THP-1–derived macrophages and Jurkat T cells (Figure 6A). After being cocultured with hsa_circ_0000652 stably expressed THP-1–derived macrophages for 24 h, T cells were separately cultured for 24 h and collected for the following analysis. As OX40 is the receptor of OX40L and TRAF2 is one of the most important downstream mediators of OX40/OX40L, we conducted qRT-PCR analysis of OX40 and TRAF2 in T cells. Expression levels of both OX40 and TRAF2 were upregulated in cells cocultured with macrophages overexpressing hsa_circ_0000652 compared to controls and were rescued by co-transfecting hsa-miR-1179 (Figure 6B, OX40: *p* < 0.001 for LV-Vector vs. LV-circ0652; *p* = 0.3525 for LV-sh-NC vs. LV-sh-circ0652; *p* = 0.0104 for miR-1179 vs. miR-1179+LV-circ0652; TRAF2: *p* < 0.001 for LV-Vector vs. LV-circ0652; *p* = 0.100 for LV-sh-NC vs. LV-sh-circ0652; *p* = 0.0258 for miR-1179 vs. miR-1179+LV-circ0652). However, knockdown of hsa_circ_0000652 in cells cocultured with macrophages did not show a statistically significant reduction in the expression of OX40 and TRAF2. These results were confirmed in the protein level analyzed by flow cytometry (Figure 6C, *p* < 0.001 for LV-Vector vs. LV-circ0652 and miR-1179 vs. miR-1179+LV-circ0652). Consistently, knockdown of hsa_circ_0000652 in macrophages resulted in restraint of OX40 expression on T cells (Figure 6D, *p* < 0.001 for LV-sh-NC vs. LV-sh-circ0652). ELISA assay showed that elevated levels of TNF-α, IL-6, and IL-23 were detected in the supernatant of T cells cocultured with macrophages overexpressing hsa_circ_0000652 and could be rescued by hsa_miR-1179 overexpression (Figures 6E–G, TNFα: *p* = 0.0015 for LV-Vector vs. LV-circ0652, *p* = 0.024 for LV-sh-NC vs. LV-sh-circ0652; *p* = 0.0215 for miR-1179 vs. miR-1179+LV-circ0652; IL-6 and IL-23: *p* < 0.001 for LV-Vector vs. LV-circ0652, LV-sh-NC vs. LV-sh-circ0652 and miR-1179 vs. miR-1179+LV-circ0652). On the contrary, knockdown of hsa_circ_0000652 in macrophages exerted adverse effects by reducing levels of cytokines mentioned above. In conclusion, hsa_circ_0000652 aggravated the inflammation of macrophages and CD4^+^ T cells *via* the hsa-miR-1179/OX40L/OX40 pathway.

## Discussion

Nowadays, patients with AS are still disturbed with misdiagnosis and mistreatment due to the lack of understanding of pathogenesis (Sieper et al., 2015; Brown et al., 2016). Emerging data show that non-coding RNAs (ncRNAs) including circRNA and miRNA, play important roles as biomarkers and regulatory factors in many rheumatic diseases (Li B. et al., 2018; Chen et al., 2019; Guo et al., 2019; Zhou et al., 2019). It has been previously reported that some circRNAs were differentially expressed in spinal ligament tissues of patients with AS (Kou et al., 2020). However, the expression profiles and functions of circRNAs in immune cells of AS are still beyond research. In the current study, we presented the differential expression profiles of circRNAs in PBMCs from AS patients by circRNA sequencing. Then we found that a circRNA hsa_circ_0000652 was upregulated in AS patients and correlated with multiple indexes of disease activity, indicating its potential to be a novel biomarker and therapeutic target. Gain-of-function and loss-of-function assays showed that hsa_circ_0000652 promoted macrophage-induced inflammation. Besides, hsa_circ_0000652 upregulated OX40L by sponging hsa-miR-1179 and aggravated the interaction between macrophages and CD4^+^ T cells. These findings uncovered the characteristics and pro-inflammatory functions of hsa_circ_0000652 and OX40L in AS.

Mounting studies show that antigen processing cells (APCs) are deeply involved in the pathogenesis of AS (Smith, 2015; Ranganathan et al., 2017b; Menegatti et al., 2020). Among APCs, macrophages are key pro-inflammatory cells in many rheumatic diseases and participate in osteoclastogenesis (Horwood, 2016; Ranganathan et al., 2017a). However, the roles of AS-associated circRNAs in the activation of macrophages remain poorly understood. Our current study demonstrated that the cell ratio of CD3^−^CD14^+^ cells was greater in AS patients, indicating that abnormal activated monocyte macrophage may be involved in the inflammation of AS. Meanwhile, hsa_circ_0000652 significantly promoted proliferation and cytokine production and inhibited the apoptosis of activated macrophages. These data suggested that hsa_circ_0000652 acted as a pro-inflammatory regulator in the proliferation and cytokine secretion in AS-associated macrophages.

In many biological processes, circRNAs are basically functional in acting as sponges in ceRNA, regulating RNA-binding proteins and DNA transcription or RNA translation, and being translated into polypeptides (Li X. et al., 2018; Kristensen et al., 2019; Lei et al., 2020). In the current study, we found that hsa_circ_0000652 bound with hsa-miR-1179, while hsa-miR-1179 bound with the 3′-UTR regions of OX40L. Through the ceRNA network, hsa_circ_0000652 positively regulated OX40L expression, which was restored by hsa-miR-1179. These data implicated that hsa_circ_0000652 upregulated the expression of OX40L by sponging hsa-miR-1179.

OX40L along with its receptor, OX40, are known as co-stimulatory molecules and have great effects on T-cell activation and cytokine production *via* the interaction with APCs(Croft, 2009; 2010). Previous studies found that OX40/OX40L is correlated with multiple rheumatic diseases and has the potential to be therapeutic targets (Laustsen et al., 2014; Jacquemin et al., 2018; Fu et al., 2020). Yet, the functions of OX40/OX40L in AS have not been investigated. Our current study illustrated that OX40L was mainly expressed on CD3^−^CD14^+^ cells and OX40 was mainly located on CD4^+^ T cells in AS. As macrophages could be derived from CD3^−^CD14^+^ cells, we speculated that OX40L expression in macrophages could be regulated by hsa_circ_0000652/hsa-miR-1179. Using the macrophage—T-cell coculture system, we discovered that hsa_circ_0000652 could enhance the interaction between macrophages and T cells by regulating OX40/OX40L. These results indicated that hsa_circ_0000652–regulated OX40/OX40L may participate in the inflammatory process in AS pathogenesis.

**FIGURE 7:**
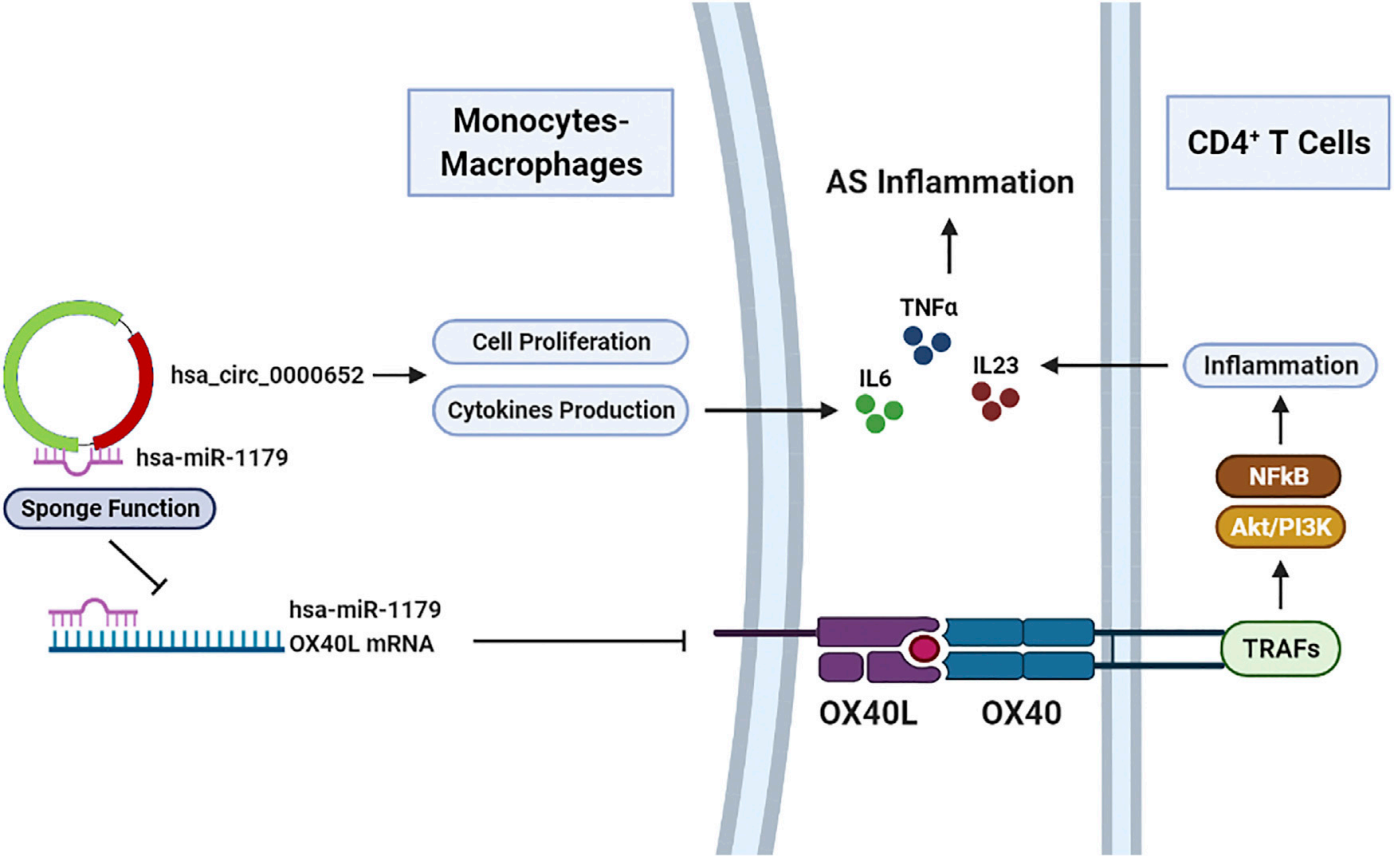
Hsa_circ_0000652 serves as a pro-inflammatory factor in macrophage activation and an enhancer of OX40/OX40L interaction between macrophages and CD4^+^ T cells.

Collectively, our findings revealed that hsa_circ_0000652 was upregulated in AS patients and aggravated the inflammation through activating macrophages and enhancing OX40/OX40L interaction by sponging hsa-miR-1179 (Figure 7). Upregulated OX40L on macrophages could further interact with OX40 on CD4^+^ T cells and reinforce inflammation. Unfortunately, due to the lack of a robust mouse model that perfectly mimics the pathogenesis of AS, *in vivo* analysis of hsa_circ_0000652 and OX40/OX40L could not be conducted in the current study. In conclusion, this study may illuminate the novel functions of hsa_circ_0000652 in the pathogenesis of AS and imply that targeting the hsa_circ_0000652/hsa-miR-1179/OX40L axis may be a promising therapeutic strategy for AS treatment.

## Data Availability

The data presented in the study are deposited in the NCBI Gene Expression Omnibus repository, accession number: GSE178408. Further inquiries can be directed to the corresponding authors.
