# Supplementary material for: Hsa_circ_0000652 Aggravates Inflammation by Activation of Macrophages and Enhancement of OX40/OX40L Interaction in Ankylosing Spondylitis
Source: Front Cell Dev Biol. 2021 Dec 16;9:737599. doi: 10.3389/fcell.2021.737599 (PMC8716807; doi:10.3389/fcell.2021.737599)
Supplement: Supplementary file 1 [file Table1.DOCX]

**Supplementary table S1**

Primers used for qRT-PCR.

| Genes | Forward (5’-3’) | Reverse (5’-3’) |
| --- | --- | --- |
| hsa_circ_0000652 | GGCTAAGCAGGACAAAATGA | AAGCATGGCATTCGGATTTT |
| IQGAP1 | AATTCCAGCCGACACATTTG | TGAATTTCAGCTTGCGTGAG |
| OX40L | ATCCTTTCCAACAAGTCATCCAGCA | CGAGGATACCGATGTGATACCATGAG |
| OX40 | TGGTGTAACCTCAGAAGTG | GTCAACTCCAGGCTTGTA |
| β-Actin | CATGTACGTTGCTATCCAGGC | CTCCTTAATGTCACGCACGAT |
| GAPDH | GGAGCGAGATCCCTCCAAAAT | GGCTGTTGTCATACTTCTCATGG |
| IL1b | AAACAGATGAAGTGCTCCTTCCAGG | TGGAGAACACCACTTGTTGCTCCA |
| IL12 | CCTTGCACTTCTGAAGAGATTGA | ACAGGGCCATCATAAAAGAGGT |
| TNFα | CCTCTCTCTAATCAGCCCTCTG | GAGGACCTGGGAGTAGATGAG |
| TRAF2 | GCTCATGCTGACCGAATGTC | GCCGTCACAAGTTAAGGGGAA |
