# Supplementary material for: Hsa_circ_0000652 Aggravates Inflammation by Activation of Macrophages and Enhancement of OX40/OX40L Interaction in Ankylosing Spondylitis
Source: Front Cell Dev Biol. 2021 Dec 16;9:737599. doi: 10.3389/fcell.2021.737599 (PMC8716807; doi:10.3389/fcell.2021.737599)
Supplement: Supplementary file 2 [file Table2.DOCX]

**Supplementary table S2**

Sequences used in this study.

|  | Sequence (5’-3’) |
| --- | --- |
| hsa_circ_0000652 FISH probe | AGCAATAACAGCAGCATGTATATTGACTTTGTTTATATTG |
| hsa-miR-1179 FISH probe | CCAACCAATGAAAGAATGCTT |
| hsa-miR-1179 mimic | AAGCAUUCUUUCAUUGGUUGG |
| hsa_circ_0000652 Wild-type | TACATGCTGCTGTTATTGCTATTAATGAAGCTATTGACCGTAGAATTCCAGCCGACACATTTGCAGCTTTGAAAAATCCGAATGCCATGCTTGTAAATCTTGAAGAGCCCTTGGCATCCACTTACCAGGATATACTTTACCAGGCTAAGCAGGACAAAATGACAAATGCTAAAAACAGGACAGAAAACTCAGAGAGAGAAAGAGATGTTTATGAGGAGCTGCTCACGCAAGCTGAAATTCAAGGCAATATAAACAAAGTCAATA |
| hsa_circ_0000652 Mutant variant | TACATGCTGCTGTTATTGCTATTAATGAAGCTATTGACCGTAGAATTCCAGCCGACACATTTGCAGCTTTGAAAAATCCGAATGCCATGCTTGTAAATCTTGAAGAGCCCTTGGCATCCACTTACCAGGATATACTTTACCAGGCTAAGCAGGACAAAATGACAAATGCTAAAAACAGGACAGAAAACTCAGAGAGAGAAAGAGATGTTTATGAGGAGCTGCTCACGCAAGCTGAAATTCAAGGCAATATAAACAAAGTCAATA |
| hsa_circ_0000652 pull-down probe | CAGCAGCATGTATATTGACT |
| hsa-miR-1179 pull-down probe | UCCAGCA+UCAGUGAUU+UUGUUG |
| sh-hsa_circ_0000652 | AGAGAGATAATTGGAATTAATTTGACTGTAAACACAAAGATATTAGTACAAAATACGTGACGTAGAAAGTAATAATTTCTTGGGTAGTTTGCAGTTTTAAAATTATGTTTTAAAATGGACTATCATATGCTTACCGTAACTTGAAAGTATTTCGATTTCTTGGCTTTATATATCTTGTGGAAAGGACGAAACACCGGAAACAGGACAGAAAACTCACTCGAGTGAGTTTTCTGTCCTGTTTTTTTTGAATTCTCGACCTCGAGACAAATGGCAGTATTCATCCACAATTTTAAAAGAAAAGGGGGGATTGGGGGGTACAGTGCAGGGGAAAGAATAGTAGACATAATAGCAACAGACATACAAACTAAAGAATTACAAAAACAAAT |
